# Draft genome sequence of *Enterococcus faecium* strain LMG 8148

**DOI:** 10.1186/s40793-016-0187-1

**Published:** 2016-09-07

**Authors:** Joran E. Michiels, Bram Van den Bergh, Maarten Fauvart, Jan Michiels

**Affiliations:** 1 Centre of Microbial and Plant Genetics, KU Leuven, B-3001 Leuven, Belgium; 2 Department of Life Science Technologies, imec, Smart Systems and Emerging Technologies Unit, B-3001 Leuven, Belgium

**Keywords:** Draft genome, Gut commensal, Nosocomial pathogen, *Enterococcus faecium*, Human isolate

## Abstract

*Enterococcus faecium*, traditionally considered a harmless gut commensal, is emerging as an important nosocomial pathogen showing increasing rates of multidrug resistance. We report the draft genome sequence of *E. faecium* strain LMG 8148, isolated in 1968 from a human in Gothenburg, Sweden. The draft genome has a total length of 2,697,490 bp, a GC-content of 38.3 %, and 2,402 predicted protein-coding sequences. The isolation of this strain predates the emergence of *E. faecium* as a nosocomial pathogen. Consequently, its genome can be useful in comparative genomic studies investigating the evolution of *E. faecium* as a pathogen.

## Introduction

Enterococci commonly reside in the gastro-intestinal tract of a wide variety of invertebrate and vertebrate hosts, including humans. Since they produce bacteriocins, *Enterococcus* spp. are widely used as starter cultures for food fermentations or probiotic supplements [1]. Since the 1970s however, they have enigmatically progressed from commensal organisms of little clinical interest to leading nosocomial pathogens causing infections of the urinary tract, bloodstream, and surgical wounds, among others [2]. The large majority of human enterococcal infections are caused by two species: *E. faecalis* and *E. faecium*. Worryingly, acquired antibiotic resistance against a multitude of drugs is increasingly being reported in these organisms [3].

Here, we report the draft genome of *E. faecium*LMG 8148, a strain of human origin isolated in 1968 in Gothenburg, Sweden [4].

## Organism information

### Classification and features

**Fig. 1:**
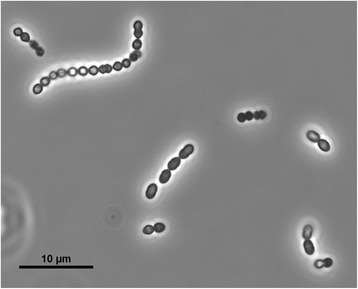
Phase-contrast micrograph of *E. faecium* LMG 8148

**Fig. 2:**
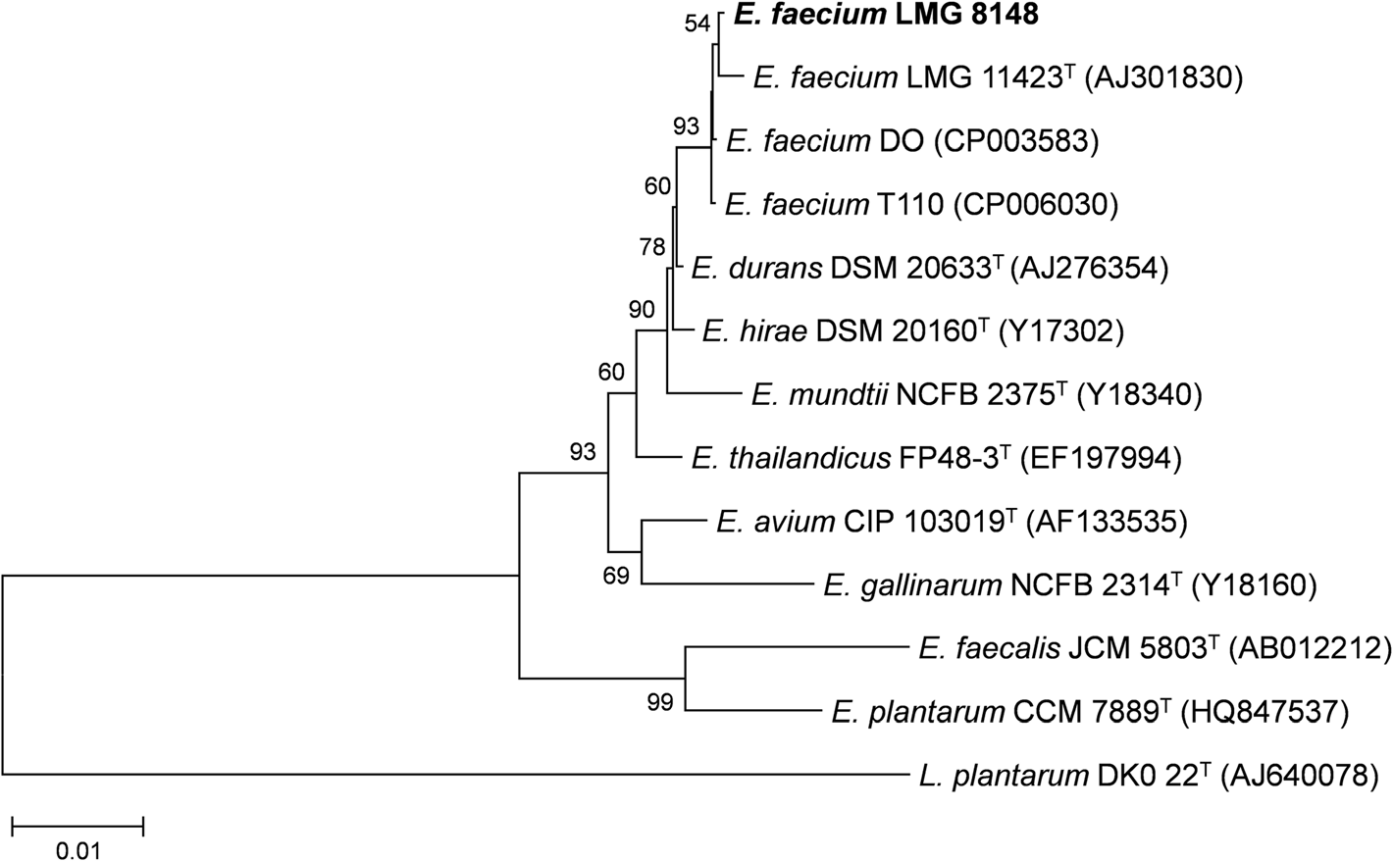
16S rRNA phylogenetic tree indicating the position of *E. faecium* LMG 8148 relative to other *E. faecium* strains and other enterococcal species (type strain = ^T^). *Lactobacillus plantarum* was included as an outgroup. Genbank accession numbers of the aligned sequences are indicated between brackets. 16S rDNA sequences were aligned using MUSCLE, and the phylogenetic tree was determined using the neighbour-joining algorithm with the Kimura 2-parameter distance model in MEGA (version 7) [27]. A gamma distribution (shape parameter = 1) was used for rate variation among sites. The optimal tree with the sum of branch lengths = 0.1983 is shown, and nodes that appeared in more than 50 % of replicate trees in the bootstrap test (1000 replicates) are marked with their bootstrap support values

*Enterococcus* is a large genus of Gram-positive, non-sporulating, facultative anaerobic, round-shaped, lactic acid-producing bacteria (Table 1) [5]. *E. faecium* belongs to the family *Enterococcaceae*, order *Lactobacillales*, class *Bacilli*, and phylum *Firmicutes*. Microscopically, enterococci are often observed as pairs or short chains of cells (Fig. 1) [5]. They were classified as group D streptococci until assigned a separate genus in 1984 [6]. *E. faecalis* and *E. faecium* are the two most prominent species within the genus. Enterococci can grow in a wide range of environmental conditions, including temperature (5-50 °C), pH (4.6-9.9), 40 % (w/v) bile salts, and 6.5 % NaCl [7]. To investigate evolutionary relationships with other *Enterococcus* species and *E. faecium* strains, a phylogenetic tree was constructed using 16S rDNA sequences (Fig. 2). As expected, *E. faecium*LMG 8148 forms a cluster with the other *E. faecium* strains.

**Table 1:** Classification and general features of *Enterococcus faecium* strain LMG 8148 according to the MIGS recommendations [8]

MIGS ID	Property	Term	Evidence code^a^
	Classification	Domain *Bacteria*	TAS [16]
		Phylum *Firmicutes*	TAS [17]
		Class *Bacilli*	TAS [18, 19]
		Order *Lactobacillales*	TAS [19, 20]
		Family *Enterococcaceae*	TAS [19, 21]
		Genus *Enterococcus*	TAS [6]
		Species *Enterococcus faecium*	TAS [6]
		Strain LMG 8148	NAS
	Gram stain	Positive	TAS [22]
	Cell shape	Coccus	TAS [22]
	Motility	Non-motile	NAS
	Sporulation	Non-sporulating	TAS [7]
	Temperature range	5-50 °C	TAS [7]
	Optimum temperature	37 °C	TAS [23]
	pH range; Optimum	4.6-9.9; 7.5	TAS [23]
	Carbon source	Glucose, citrate, complex carbon sources	TAS [24, 25]
MIGS-6	Habitat	Gastro-intestinal tracts of humans and other mammals	TAS [5]
MIGS-6.3	Salinity	0-6.5 %	TAS [7]
MIGS-22	Oxygen requirement	Facultatively anaerobic	TAS [7]
MIGS-15	Biotic relationship	Commensal	TAS [5]
MIGS-14	Pathogenicity	Pathogenic	TAS [5]
MIGS-4	Geographic location	Sweden	NAS
MIGS-5	Sample collection	1961	TAS [4]
MIGS-4.1	Latitude	Unknown	NAS
MIGS-4.2	Longitude	Unknown	NAS
MIGS-4.4	Altitude	Unknown	NAS

^a^Evidence codes - *IDA* inferred from direct assay, *TAS* traceable author statement (i.e., a direct report exists in the literature); NAS: Non-traceable VAuthor Statement (i.e., not directly observed for the living, isolated sample, but based on a generally accepted property for the species, or anecdotal evidence). These evidence codes are from the Gene Ontology project [26]

## Genome sequencing information

### Genome project history

The strain LMG 8148 was isolated from a human in Gothenburg (Sweden) in 1968 [4]. The strain was obtained through the Belgian Coordinated Collection of Microorganisms. DNA samples were sequenced at the EMBL GeneCore facility (Heidelberg, Germany) and assembled using CLC Genomics Workbench (version 7.5.1). The draft genome was annotated using the NCBI Prokaryotic Genome Annotation Pipeline. This draft whole-genome sequence has been deposited at DDBJ/ENA/GenBank under the accession LOHT00000000. The project information, and its association with MIGS version 2.0 [8], is summarised in Table 2.

**Table 2:** Project information

MIGS-ID	Property	Term
MIGS-31	Finishing quality	High-quality draft
MIGS-28	Libraries used	One paired-end Illumina library (Nextera)
MIGS-29	Sequencing platforms	Illumina HiSeq 2000
MIGS-31.2	Fold coverage	317
MIGS-30	Assemblers	CLC NGS Cell 7.5.1
MIGS-32	Gene calling method	GeneMarkS+
	Locus Tag	AUC59
	Genbank ID	LOHT00000000
	GenBank Date of Release	2016/02/26
	GOLD ID	-
	BIOPROJECT	PRJNA305395
MIGS-13	Source Material Identifier	LMG 8148
	Project relevance	Evolution

### Growth conditions and genomic DNA preparation

Bacterial cultures were inoculated from single colonies on lysogeny broth agar in 5 ml of lysogeny broth and grown overnight at 37 °C, with 200 rpm orbital shaking. The DNeasy Blood&Tissue Kit (Qiagen) was used for DNA isolation, following the manufacturer’s instructions and pre-treatment protocol for Gram-positive bacteria. Concentration and purity of isolated DNA was determined spectrophotometrically using the Nanodrop ND-1000 and fluorometrically using Qubit analysis (ThermoFisher Scientific).

### Genome sequencing and assembly

100 bp paired-end sequencing was performed on an Illumina HiSeq 2000 machine at the EMBL GeneCore facility in Heidelberg (Germany). The total number of paired reads was 9,317,630. Sequencing data was analysed with the Qiagen CLC Genomics workbench version 7.5.1. After a trimming step for quality (score limit: 0.05) and ambiguous nucleotides (maximum 2 ambiguities), reads were assembled *de novo* using a mismatch cost of 2, a deletion cost of 3, an insertion cost of 3, length fraction 0.5, and similarity fraction 0.8. The assembly yielded 366 contigs (minimum length 200 bp) with an average coverage of 317× and an average contig length of 7,370 bp (N50 length of 41,184 bp). The total length of the draft genome is 2,697,490 bp with a GC-content of 38.3 %.

### Genome annotation

All contigs were annotated using NCBI’s Prokaryotic Genome Annotation Pipeline. Pfam domains [9] in the predicted protein sequences were identified using the Batch Web CD-Search Tool from NCBI [10]. Predicted proteins were classified into COG [11] functional categories using the WebMGA web server for metagenomic analysis [12]. For further characterization of the predicted genes, CRISPRFinder [13], the SignalP 4.1 server [14], and the TMHMM server [15] were used to predict CRISPR repeats, signal peptides, and transmembrane domains, respectively. For the CRISPRFinder tool, only confirmed CRISPRs and not questionable CRISPRs were taken into account.

## Genome properties

The properties of this draft genome are summarised in Table 3. Assembly yielded 366 contigs containing 2,697,490 bp with a 38.3 % GC-content. The total number of 2,772 genes predicted by PGAP includes 2,402 protein coding genes (totalling 2,136,945 base pairs), 303 pseudo genes, and 67 RNA genes (56 tRNA and 11 rRNA genes). For 19.37 % of the protein-coding genes, no putative function was assigned, and these were annotated as hypothetical proteins. Further characteristics of the predicted genes are given in Table 3, and classification into functional COG categories is shown in Table 4.

**Table 3:** Genome statistics

Attribute	Value	% of Total
Genome size (bp)	2,697,490	100.00
DNA coding (bp)	2,136,945	79.22
DNA G + C (bp)	1,034,256	38.34
DNA scaffolds	366	100.00
Total genes	2,772	100.00
Protein coding genes	2,402	86.65
RNA genes	67	2.42
Pseudo genes	303	10.93
Genes in internal clusters	-	-
Genes with function prediction	2,235	80.63
Genes assigned to COGs	2,153	77.67
Genes with Pfam domains	2,078	74.96
Genes with signal peptides	120	4.33
Genes with transmembrane helices	631	22.76
CRISPR repeats	1	-

**Table 4:** Number of genes associated with general COG functional categories

Code	Value	%age	Description
J	150	6.24	Translation, ribosomal structure and biogenesis
A	0	0.00	RNA processing and modification
K	185	7.70	Transcription
L	148	6.16	Replication, recombination and repair
B	0	0.00	Chromatin structure and dynamics
D	21	0.87	Cell cycle control, cell division, chromosome partitioning
V	49	2.04	Defense mechanisms
T	88	3.66	Signal transduction mechanisms
M	114	4.75	Cell wall/membrane biogenesis
N	13	0.54	Cell motility
U	27	1.12	Intracellular trafficking and secretion
O	58	2.41	Posttranslational modification, protein turnover, chaperones
C	74	3.08	Energy production and conversion
G	253	10.53	Carbohydrate transport and metabolism
E	144	6.00	Amino acid transport and metabolism
F	78	3.25	Nucleotide transport and metabolism
H	55	2.29	Coenzyme transport and metabolism
I	57	2.37	Lipid transport and metabolism
P	109	4.54	Inorganic ion transport and metabolism
Q	22	0.92	Secondary metabolites biosynthesis, transport and catabolism
R	263	10.95	General function prediction only
S	245	10.20	Function unknown
-	249	10.37	Not in COGs

The total is based on the total number of protein coding genes in the genome

## Conclusions

The presented genome sequence is from a strain isolated in 1968, and thus precedes the emergence of enterococci as important causative agents of hospital-acquired infections in the 1970s and 1980s [2]. Consequently, this genome could be useful for comparative genomic studies looking to solve the remarkable recent emergence of *E. faecium* as a notorious nosocomial pathogen.

## Abbreviations

COG: Clusters of Orthologous Groups
PGAP: Prokaryotic genome annotation pipeline
